# Multimerization- and glycosylation-dependent receptor binding of SARS-CoV-2 spike proteins

**DOI:** 10.1371/journal.ppat.1009282

**Published:** 2021-02-08

**Authors:** Kim M. Bouwman, Ilhan Tomris, Hannah L. Turner, Roosmarijn van der Woude, Tatiana M. Shamorkina, Gerlof P. Bosman, Barry Rockx, Sander Herfst, Joost Snijder, Bart L. Haagmans, Andrew B. Ward, Geert-Jan Boons, Robert P. de Vries

**Affiliations:** 1 Department of Chemical Biology & Drug Discovery, Utrecht Institute for Pharmaceutical Sciences, Utrecht University, Utrecht, The Netherlands; 2 Department of Integrative Structural and Computational Biology, The Scripps Research Institute, La Jolla, California, United States of America; 3 Biomolecular Mass Spectrometry and Proteomics, Department of Chemistry, Faculty of Science, Utrecht University, Utrecht, The Netherlands; 4 Bijvoet Center for Biomolecular Research, Utrecht University, Utrecht, The Netherlands; 5 Department of Viroscience, Erasmus University Medical Center, Rotterdam, The Netherlands; 6 Complex Carbohydrate Research Center, University of Georgia, Athens, Georgia, United States of America; 7 Department of Chemistry, University of Georgia, Athens, Georgia, United States of America; Johns Hopkins University Bloomberg School of Public Health, UNITED STATES

## Abstract

Receptor binding studies on sarbecoviruses would benefit from an available toolkit of recombinant spike proteins, or domains thereof, that recapitulate receptor binding properties of native viruses. We hypothesized that trimeric Receptor Binding Domain (RBD) proteins would be suitable candidates to study receptor binding properties of SARS-CoV-1 and -2. Here we created monomeric and trimeric fluorescent RBD proteins, derived from adherent HEK293T, as well as in GnTI-/- mutant cells, to analyze the effect of complex vs high mannose glycosylation on receptor binding. The results demonstrate that trimeric, complex glycosylated proteins are superior in receptor binding compared to monomeric and immaturely glycosylated variants. Although differences in binding to commonly used cell lines were minimal between the different RBD preparations, substantial differences were observed when respiratory tissues of experimental animals were stained. The RBD trimers demonstrated distinct ACE2 expression profiles in bronchiolar ducts and confirmed the higher binding affinity of SARS-CoV-2 over SARS-CoV-1. Our results show that complex glycosylated trimeric RBD proteins are attractive to analyze sarbecovirus receptor binding and explore ACE2 expression profiles in tissues.

## Author summary

The ongoing COVID19 pandemic necessitates additional tools to study SARS-CoV receptor binding properties. This increases our understanding of how these viruses attach to cells, essential to monitor possible adaptations and determine the zoonotic capabilities of these viruses. Here we created fluorescent multimeric RBD proteins with two distinct glycosylation profiles. The results demonstrate that trimeric fully glycosylated RBD trimers are excellent tools to study receptor binding properties.

## Introduction

SARS-CoV-2 has sparked a society changing pandemic, and additional means to understand this virus will facilitate counter-measures. SARS coronaviruses carry a single protruding envelope protein, called spike, that is essential for binding to and subsequent infection of the host cell. The SARS coronavirus trimeric spike protein consists of 3 protomers of about 140kD each, containing 60 N-linked glycosylation sites. The spike is composed of an S1 and S2 domain, in which S2 contains membrane fusion activity. S1 of coronaviruses can be further divided into N-terminal and C-terminal domains (NTD & CTD), which both can contain the receptor-binding domain, depending on the viral genus. In SARS-CoV-1 and -2 this domain is located in the CTD and referred to as the receptor-binding domain (RBD). The RBD binds to angiotensin-converting enzyme 2 (ACE2) [1–3], which functions as an entry receptor for SARS-CoV. After binding and internalization, several proteinases induce the spike protein into its fusogenic form allowing the fusion of the viral and target membrane. Although this pathway is known, the details that are of importance for receptor binding and what differentiates SARS-CoV-2 from SARS-CoV-1, are incompletely understood. It has been shown that the affinity of the SARS-CoV-2 spike to ACE2 is significantly higher compared to SARS-CoV-1 [2,4]. However, how this relates to tissue and cell tropism remains to be determined.

Several recombinant protein approaches to create coronavirus spike proteins have been utilized with success for the development of serological assays [5], elucidating the spike structures, and isolation of neutralizing antibodies [6,7]. However, the conformation of the receptor-binding domain of the spike, which can be in the “up” and “down” configuration [8–12], is highly variable. This variability is important for receptor binding as only in the “up” conformation the RBD can bind ACE2. Approaches to control the conformation for vaccine purposes using stabilization mutations appear to keep the RBD conformation in their down-state [11,12], making them non preferred proteins to analyze receptor-binding properties. Also, the multimerization status of the RBD is critical to allow for correct analysis of ACE2 interactions. While monomeric RBD proteins can be efficiently made in large quantities [5], and bind ACE2, they are hardly used in receptor binding assays to cells and tissues. The majority of studies analyzing RBD protein binding to cells and tissues, utilize Fc-tagged proteins [2]. Fc-tags on recombinant proteins are extremely convenient for mammalian cell expression and purification systems, which result in dimeric recombinant spikes that are biologically functional. However, the native coronavirus spike is trimeric and thus Fc tagged spikes do not fully recapitulate native properties.

We hypothesized that fluorescent trimeric RBD proteins would provide complementary means to study RBD-receptor interactions. In this study, we compared monomeric and trimeric SARS-CoV RBD with full-length trimeric spikes expressed in cells producing proteins with either complex or high mannose glycosylation. The fusion of sfGFP and mOrange2 at the C-terminus has previously been shown to increase expression yields, protein stability, and afford additional means for fluorescent-based experiments [13], and thus are attractive to be fused to RBD proteins. The resulting proteins were analyzed for binding to cell culture cells and paraffin-embedded tissues of various hosts including susceptible and non-susceptible animals. The results demonstrate that fully glycosylated trimeric SARS-CoV-2 RBD proteins reveal the differences in ACE2 expression between cell cultures and tissue sections. These trimeric RBD proteins bind ACE2 efficiently in a species-dependent manner and can be used to profile ACE2 tissue expression. Finally, we observed distinct expression of ACE2 in bronchioles of experimental animal models.

## Results

### Generation of fluorescent coronavirus receptor-binding domain spike proteins

**Fig 1:**
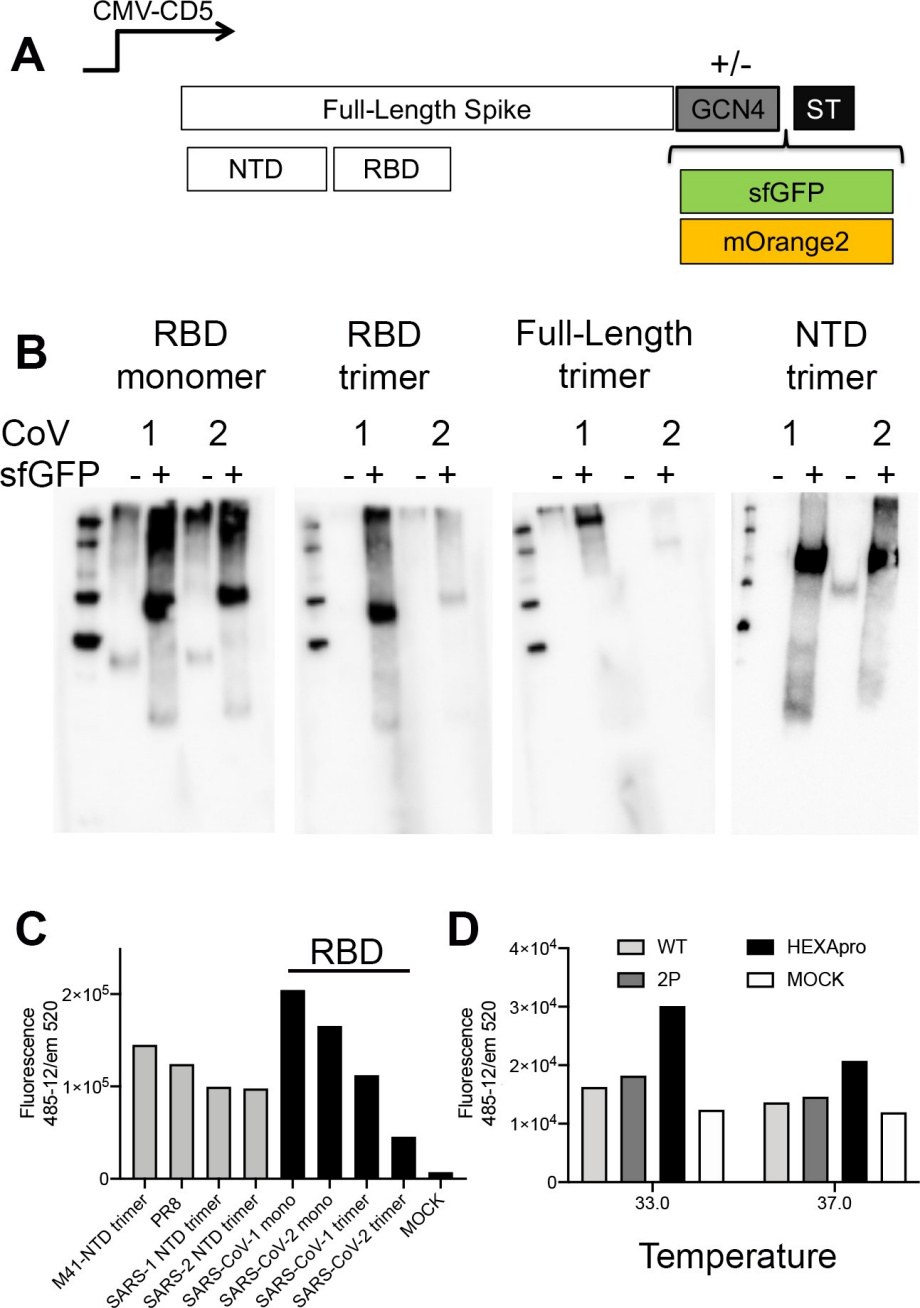
Expression of coronavirus spike proteins using sfGFP and mOrange2 fusions. **(A) Spike expression plasmids with and without GCN4 trimerization motif fused with either sfGFP or mOrange2.** Schematic representation of the used spike expression cassette. The spike open reading frame is under the control of a CMV-promotor and was cloned in frame with DNA sequences coding for the CD5 signal peptide. At the C-terminus a GCN4 trimerization domain followed by sfGFP or mOrange2, and a TEV cleavable Strep-tag II. **(B) Expression analyses of non and sfGFP fused monomeric and trimeric RBD, the full-length ectodomain spike, and the NTD proteins.** Denatured samples of the cell culture supernatant were subjected to SDS-PAGE and western blot analyzes stained with an anti-Streptag-HRP antibody. **(C) Quantification.** sfGFP emission was directly measured in the supernatants. **(D) Full-length spike adaptation to the hexapro variant.** Full-length spike expression vectors containing the wildtype, 2P, or Hexapro were expressed at 33 or 37C and fluorescence was measured in cell culture supernatant 4 days post-transfection.

To create recombinant, fluorescent, soluble, full-length ectodomains, NTDs, and RBDs, we cloned these open reading frames (ORF) in plasmids with and without the GCN4 trimerization domain, fused to either sfGFP or mOrange (Fig 1A). The monomeric and trimeric RBDs were efficiently expressed in both HEK 293T, a commonly used cell line for recombinant expression of soluble proteins, as well as GnTI^-/-^ cells, that lack mannosidase I resulting in N-glycosylation that is stalled at Man-5. The latter is commonly used for crystallization purposes, however mature vs Man-5 protein N-glycosylation can have marked effects on different biological properties [14–16]. The addition of the sfGFP domain resulted in an increased expression yield up to 2- to 5-fold. (Fig 1B), and expression yields of the mOrange2 fusions were comparable. To illustrate the expression yields of SARS spike proteins or domains thereof we measured the fluorescence in the cell culture supernatant (Fig 1C). The wild-type full-length ectodomains were difficult to express even with the addition of sfGFP or mOrange2 fusion (Fig 1B). To increase yields for the full-length ectodomain we introduced the 2P and additional hexapro mutations [17], and analyzed the fluorescence in cell culture supernatants five days post-transfection after incubation at 33 or 37°C. Although we did not observe a large increase in yields, we were able to purify sufficient protein to compare full-length ectodomain trimers vs monomeric and trimeric RBD and NTD proteins.

### Spike RBD domains in frame with a C-terminal GCN4 and fluorescent reporter protein display multimeric features on gel and maintain antigenicity

**Fig 2:**
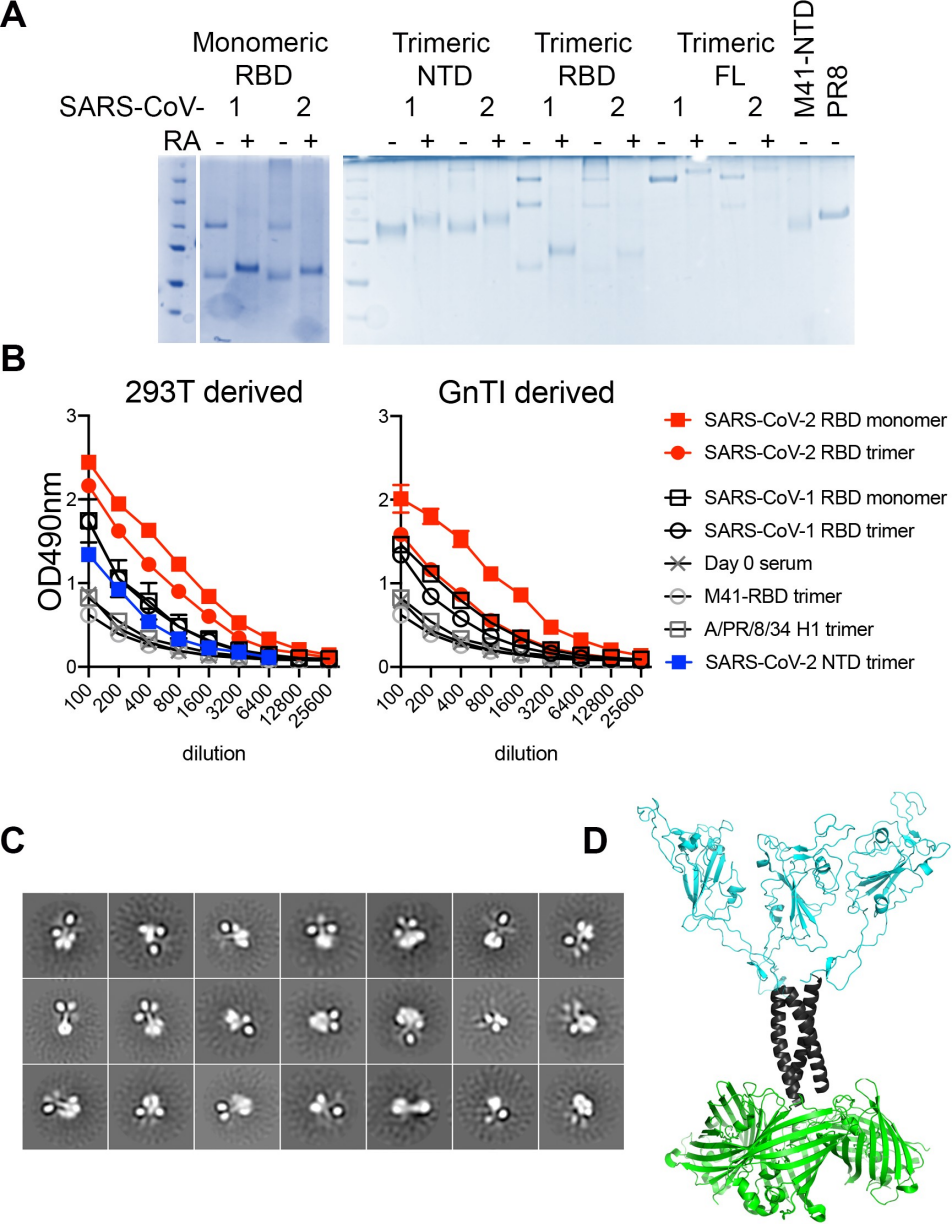
Molecular analyses. **(A) SDS-PAGE analyses of purified RBD proteins.** 500 ng of purified proteins were loaded on a gel with or without preheating for 30 minutes at 98C in the presence of a reducing agent. **(B) Antigenic analyses using 21-day post-infection macaque serum.** 2μg/ml SARS-CoV-RBD proteins were coated on 96-well plates. Proteins were detected with macaque serum for 2hrs at RT. An anti-human IgG-HRP was used to detect RBD specific antibodies. Both 293T and GnTI^-/-^ derived proteins were analyzed. As negative controls, IBV-M41-NTD and HA-PR8D were included. **(C) Negative stain EM of trimeric RBD fused to sfGFP.** Negative-stain 2D class averages of soluble RBD proteins demonstrate that they are well-folded trimers. The C-terminal helices and fusion proteins are visible in some class averages. **(D) Structural model.** Based on the crystal structure of a SARS-CoV-2 RBD in the up conformation, with a GNC4 trimerization domain with 3 sfGFP domains added.

After purification, all RBD proteins were analyzed on gel under reducing and non-reducing conditions, i.e. with or without the addition of DTT (Fig 2A). Under non-reducing conditions, constructs for monomeric RBD proteins contained an additional dimeric fraction, which could be reduced to a single monomeric form with the addition of DTT. The constructs for NTD trimers exhibited a single monomeric band on SDS-PAGE under both reducing and non-reducing conditions, indicating that no intermolecular disulfide bridges were formed. The constructs for trimeric RBD variants, on the other hand, revealed dimers and trimers under non-reducing conditions that could be converted to a single monomer form upon the addition of DTT. The NTD of prototypical γ-coronavirus IBV-M41 and influenza A virus PR8 HA were included as control proteins, neither of which exhibited higher-order oligomers under non-reducing conditions. Finally, we determined the extent of N-glycosylation maturation on purified proteins expressed in either GnTI^-/-^ or HEK293T by subjecting the monomeric and trimeric proteins to PNGaseF and EndoH treatment (S1A–S1C Fig). Upon PNGaseF treatment, all N-glycans were trimmed whereas 293T derived proteins were insensitive to EndoH, as expected. Surprisingly, trimeric RBDs derived from GnTI^-/-^ cells were partially resistant to EndoH treatment, whereas the monomers derived from GnTI^-/-^ cells appeared fully deglycosylated using EndoH. This indicated the presence of a fraction of complex glycans in the GnTI^-/-^ derived materials. We confirmed the presence of complex glycans at all individual sites in both SARS-CoV-1 and -2 RBD constructs by site-specific glycoproteomics experiments (S1D Fig and S1 Data). RBD samples were digested into glycopeptides and analyzed by LC-MS/MS with electron transfer high-energy collision dissociation (EThcD) for identification (S1 Data) [18]. Whereas the majority of all sites carried the expected GlcNAc (2)Man(5) glycans, up to one-fifth of the observed signal per site corresponded to complex glycans. Upon EndoH treatment, the fraction of GlcNAc(2)Man(5) was almost completely converted to GlcNAc(1), but the complex glycans remained undigested.

### Fluorescent multimeric Spike RBD proteins maintain antigenicity

Next, we examined the antigenicity of the SARS-CoV-1 and -2 proteins using serum collected from macaques 21 days post-infection with SARS-CoV-2 [19]. Both SARS-CoV-2 RBD monomers and trimers derived from 293T cells were efficiently recognized, indicating proper folding (Fig 2B). As expected, cross-reactivity with SARS-CoV-1 RBD was observed, yet at minimal levels, and the negative controls M41 NTD and PR8 HA displayed baseline binding, identical to pre-infection serum. The NTD trimers were likewise minimally recognized by the serum, indicating that the majority of antibodies in naïve animals after infection are directed against the SARS-CoV RBD [20]. Similar results were obtained using GnTI^-/—^derived proteins, with the RBD trimer being less efficiently recognized by the macaque serum than its monomeric counterpart. This is in line with recent observations that insect cell-derived proteins are less well bound by serum antibodies [5], indicating the importance of mature N-glycans.

### RBD fused to GCN4 and sfGFP fold as trimers with three RBD and sfGFP molecules divided by the trimerization coiled-coil

To determine whether the fluorescent RBD trimers are indeed structured in a trimeric manner we subjected these proteins to negative stain single-particle EM. The EM data revealed that the RBD proteins form stable trimers that resemble known spike structures (Fig 2C). Initially, 58,018 individual particles were picked, placed into a stack, and submitted to reference-free two-dimensional (2D) classification. From the initial 2D classes, particles that did not resemble RBD were removed, resulting in a final stack of 32,152 particles, which were then subject to Relion 2D classification. All resultant classes demonstrated evident and distinct trimeric RBD, GCN4, and three sfGFP protein structures that could be identified in the EM images. From the EM images, we generated a model in which we took the crystal structures of sfGFP, the GCN4 trimerization domain (PDB:2O7H), and the SARS-CoV-2 RBD (PDB: 6XM4) to demonstrate the likely structure of our RBD trimer (Fig 2D).

### Fluorescent multimeric Spike RBD proteins bind cell lines in an ACE2 dependent manner and similar to the full-length ectodomain

**Fig 3:**
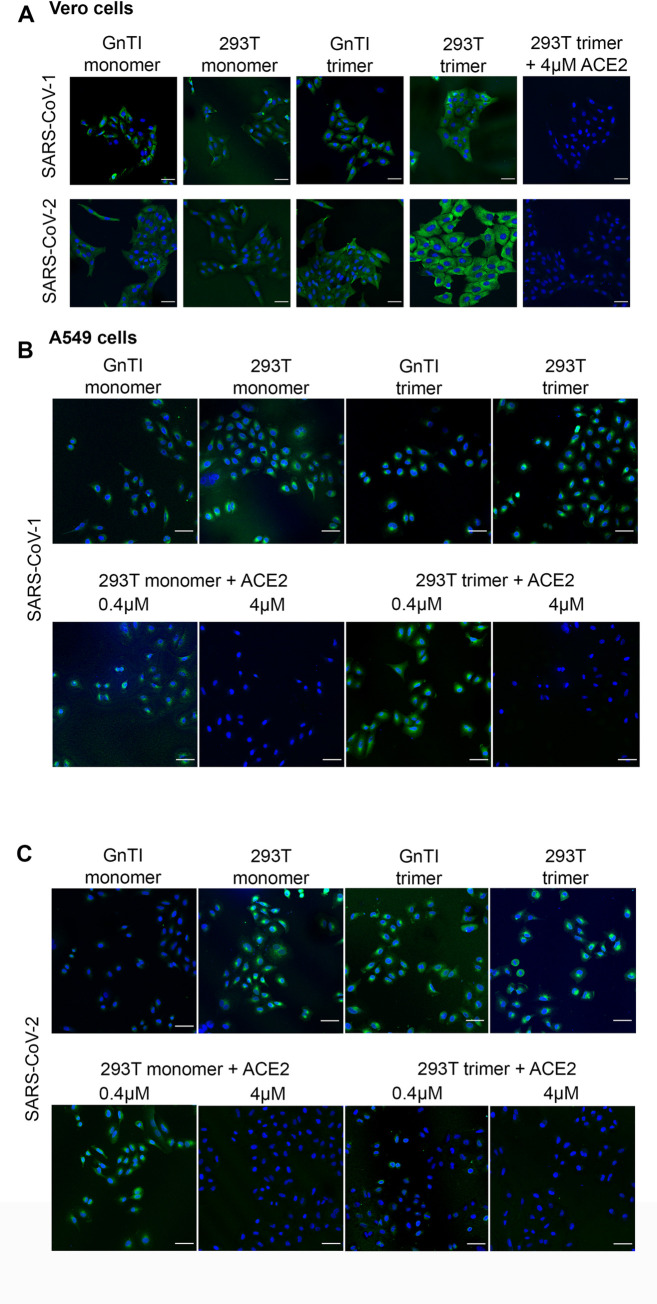
Binding of RBD proteins to VERO and A549 cells. (A) SARS-CoV-RBD proteins were applied at 50μg/ml onto VERO cells and where indicated pre-incubated with recombinant ACE2 protein. (B) Same for SARS-CoV-1 on A549 cells. (C) Same for SARS-CoV-2. SARS-CoV proteins were detected using an anti-Streptag and a goat-anti-mouse antibody sequentially. sfGFP fused SARS-CoV-RBD proteins were applied from GnTI^-/-^ monomers to 293T trimers, left to right. Scalebar is 50μm.

To determine the biological activity of our RBD proteins we stained VERO and A549 cells, which are both reported to support SARS-CoV replication with different efficiencies, with VERO cells being more susceptible compared to A549 cells [21]. On VERO cells we observed binding for all our protein preparations with an increased intensity of fully complex glycosylated SARS-CoV-2 RBD trimer compared to monomers, GNTI^-/-^ derived proteins, and SARS-CoV-1 preparations (Fig 3A). We observed no binding using the M41 receptor binding domain and an antibody only control (S2A Fig). VERO cell engagement was ACE2 dependent, as we were able to block binding using 4μM recombinant ACE2. Although A549 cells are commonly supplemented with ectopic ACE2 to support efficient SARS-CoV infection, we did observe binding of our RBD preparations (Fig 3B & 3C). Importantly, trimeric 293T-derived RBD binding was efficiently blocked using 4μM recombinant ACE2, indicating ACE2 dependency, whereas 400nM ACE2 pre-incubation was not sufficient to completely prevent binding. SARS-CoV-2 RBD proteins bound slightly more intensely to A549 cells compared to the same SARS-CoV-1 RBD proteins. Importantly, the full-length ectodomain also bound efficiently to A549 cells (S2B Fig). We did not observe any binding of the trimeric NTDs to A549 cells (S2B Fig). MDCK cells, derived from canine kidney, served as negative controls, to which we indeed did not observe any binding with any of the indicated proteins (S2C Fig). Although A549 cells need an ectopically expressed ACE2 protein to support SARS-CoV-2 virus infection, they appear to endogenously express ACE2 at levels sufficient to be bound by our RBD preparations, as it was blocked by the addition of recombinant ACE2. When we tried to confirm ACE2 expression using two different antibodies we noticed a significant difference between two antibodies obtained from Abcam (S3 Fig). However, ACE2 was present as demonstrated with the 15348 antibody in both A549 and VERO cells, indicating their susceptibility for a wide range of SARS-CoV viruses [22,23]. MDCK cells were ACE2 negative, as expected from the literature [24]. Next, we wanted to analyze receptor binding properties in a natural state using lung tissue slides from different animal species.

### Fluorescent RBD proteins fail to bind mouse, but reveal strong binding to bronchioles in ferret and Syrian hamster lung tissues

**Fig 4:**
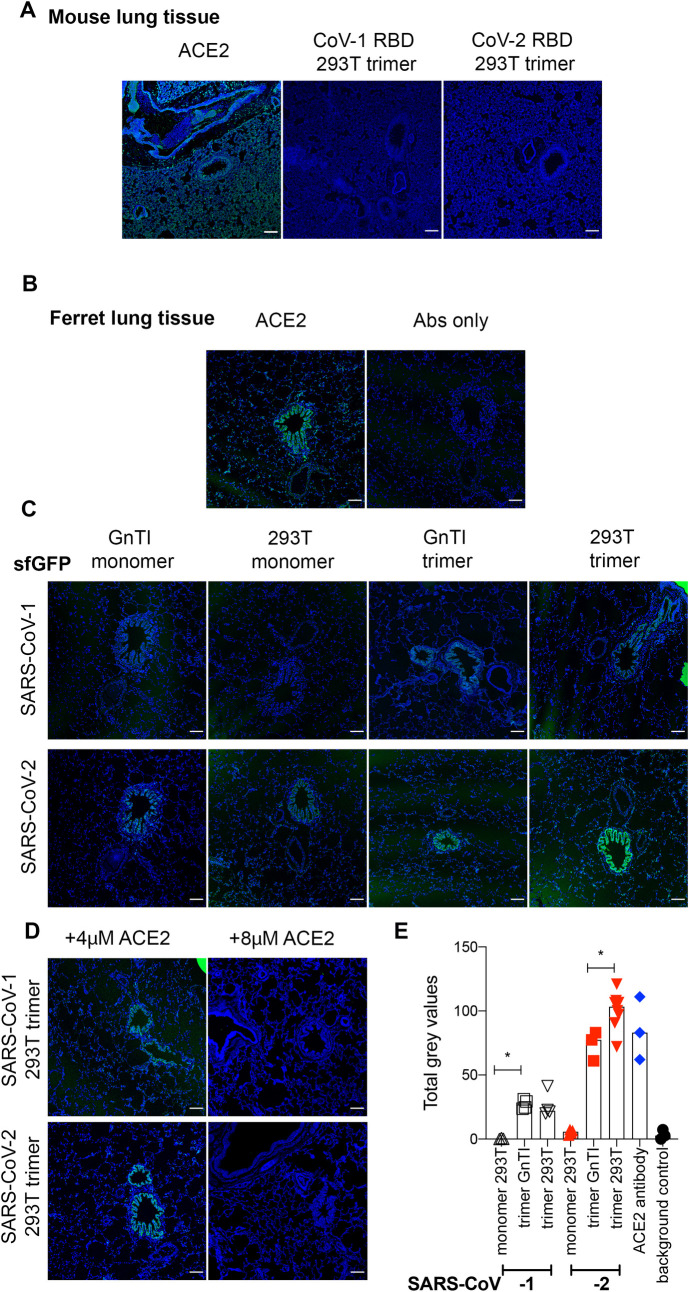
Binding of SARS-COV-RBD proteins and ACE2 antibody to mouse and ferret lung serial tissue slides. **(A) ACE2 antibody, SARS-CoV-1, and -2 on mouse lung tissue slides.** Scalebar is 100μm. **(B) ACE2 antibody and antibody only control on ferret lung tissue slides.** Scalebar is 100μm. **(C) SARS-CoV-RBD fluorescent protein localization in ferret lung tissue.** SARS-CoV-RBD proteins were applied at 50μg/ml and detected using an anti-Streptag and goat-anti-mouse antibodies sequentially. DAPI was used as a nucleic stain. Scalebar is 100μm. **(D) SARS-CoV-RBD trimer produced in HEK 293T cells pre-incubated with recombinant ACE2 before application on ferret lung tissue slides. (E) Quantification.** The intensity of gray pixels of stained ferret lung tissue slides was measured with ImageJ version 1.52p. * indicates P<0.05 as determined by an unpaired two-tailed students T-test in the GraphPad software.

Ferrets are a susceptible animal model for SARS-CoV-2 [25,26] and closely related minks are easily infected on farms [27]. Mice, on the other hand, need an ACE2 genetic knock-in to support SARS-CoV-2 infection [28]. Formalin-fixed, paraffin-embedded lung tissue slides, will most closely resemble the complex membrane structures to which spike proteins need to bind. First, ACE2 expression was assessed using an ACE2 antibody which allowed for comparisons with SARS-CoV-RBD protein binding localization. Although an antibody against human ACE2 bound significantly to the mouse lung slides, our fully glycosylated RBD trimers failed to bind, indicating a mismatch for SARS-CoV RBD to mouse ACE2 (Fig 4A). ACE2 detection in ferret lung tissue sections displayed binding to terminal bronchioles and alveoli (Fig 4B). In ferret lung tissues we determined the binding properties and strength using our different RBD preparations. We observed only minimal binding of monomeric RBD proteins derived from GnTI^-/-^ cells (Fig 4B), whereas the fully complex glycosylated counterparts showed a slight increase in fluorescent intensity, which was further increased when fully complex glycosylated trimeric RBDs were applied. In all cases, SARS-CoV-2 displayed a higher avidity compared to SARS-CoV-1. A similar trend of binding intensities was observed for monomeric, trimeric, and different N-glycosylated SARS-CoV-RBD proteins fused to mOrange2 (S4A Fig). Again specific binding was seen to the epithelium of terminal bronchioles and, to a much lower extent, to alveoli and endothelium. The results were confirmed using horseradish peroxidase readout with a hematoxylin counterstain (S4B Fig), which output is enzyme driven and purely qualitative, however, we did observe similar differences in staining intensities. Here, very minimal staining using the SARS-CoV NTD domains was observed (S4B Fig), which we did not detect using a fluorescent readout (S4C Fig). To determine if the binding was ACE2 dependent we pre-incubated trimeric RBD proteins with recombinant ACE2. While 4 μM was sufficient to block binding to cell culture cells (Fig 4C), 8 μM was needed to prevent all detectable binding to ferret lung tissue.

To confirm our observations of different binding on tissues, we quantified the intensities of the ACE2 antibody and SARS-CoV-1 and -2 RBD proteins, except for the monomeric GnTI^-/-^ derived proteins as these were almost in the background (Fig 4D). As expected a noteworthy trend was observed of increasing binding strength from SARS-CoV-1 RBD, GnTI^-/-^ derived monomers to SARS-CoV-2 fully complex glycosylated RBD trimers. Interestingly, multimerization appears to be more important for strong ACE2 interaction to tissue compared to the glycosylation status.

**Fig 5:**
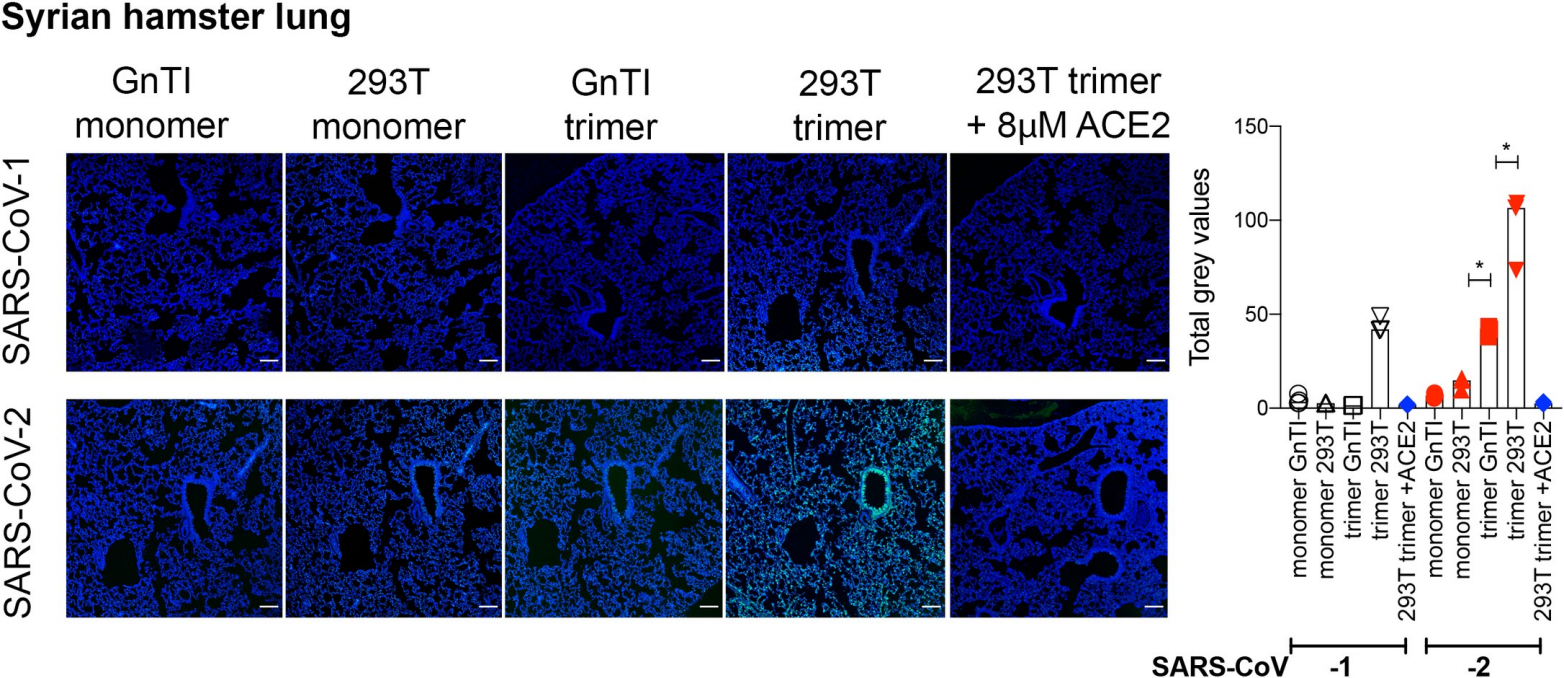
Binding of SARS-CoV-RBD proteins to serial Syrian hamster tissue slides. SARS-COV-RBD proteins (50μg/ml) were applied to Syrian hamster lung tissue slides and detected using additional anti-Streptag and goat-anti-mouse antibodies sequentially. Where indicated SARS-CoV-RBD proteins were pre-incubated with recombinant ACE2 protein. Scalebar is 100μM. For the quantification, the intensity of gray pixels of stained Syrian Hamster lung tissue slides was measured with ImageJ version 1.52p. * indicates P<0.05 as determined by an unpaired two-tailed students T-test in the GraphPad software.

Next, we applied our RBD proteins on the lung tissue of Syrian hamsters, which are currently the preferred small animal model and known to be highly susceptible to SARS-CoV-1 and -2 [29–31]. Here again, trimeric fully complex glycosylated RBD proteins were superior in binding compared to the Man-5 containing and monomeric counterparts. SARS-CoV-2 bound with significantly higher intensity to terminal bronchioles compared to SARS-CoV-1 (Fig 5). Importantly, all binding could be inhibited by pre-incubation of the RBD proteins with recombinant ACE2, indicating that binding intensity differences are likely ACE2 related.

### SARS-CoV binding completely overlaps with ACE2, which is recognized by the antibody at 4 days post-infection in Syrian hamster, but not by fluorescent RBD trimers

**Fig 6:**
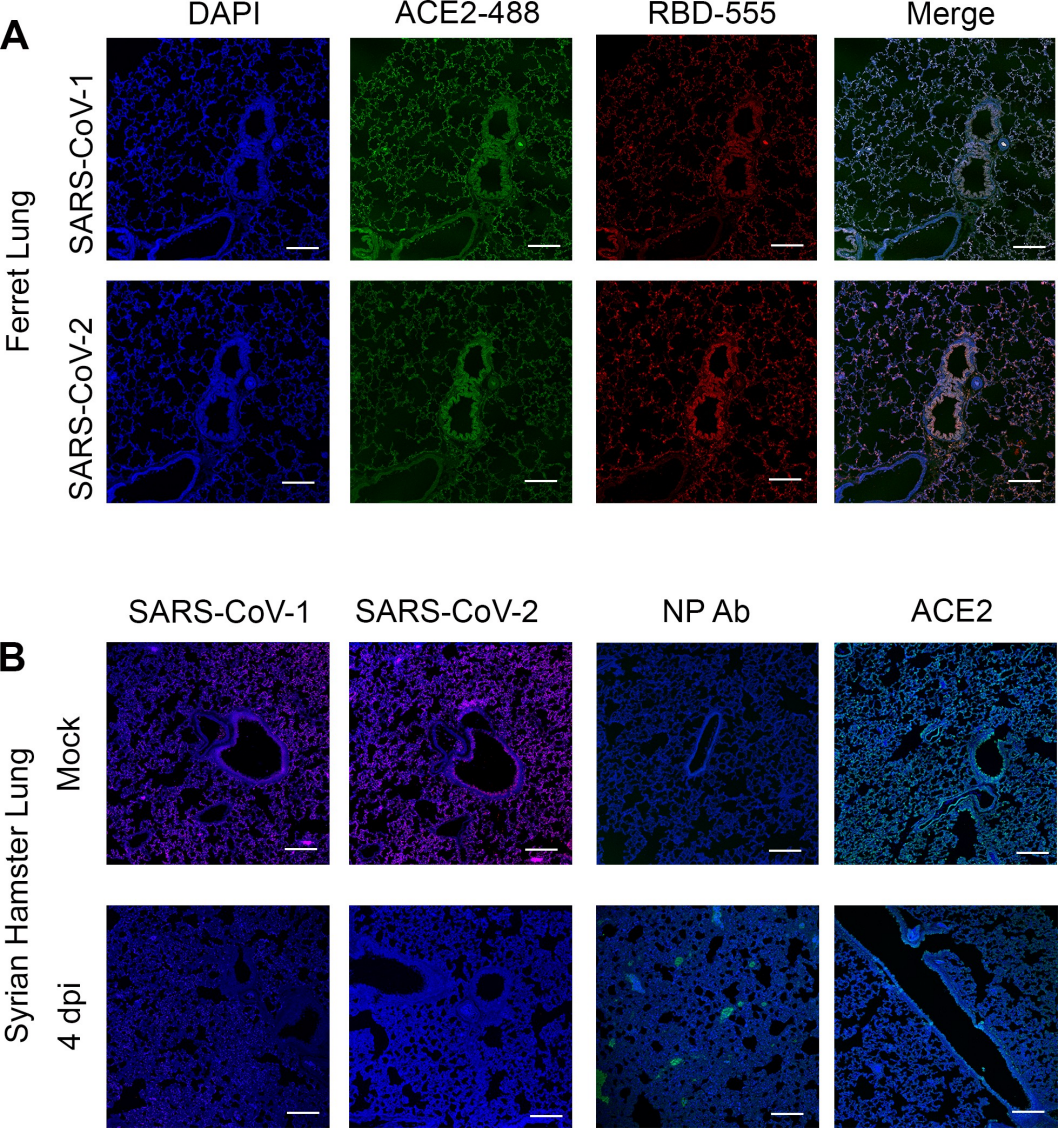
**(A) ACE2 antibody, SARS-CoV-1, and -2 co-stainings on ferret lung tissue slides.** Scalebar is 100μm. **(B) ACE2 antibody, antibody only control, and SARS-CoV-1 and -2 on Syrian hamster tissue slide from mock and infected animals 4 days post-infection.** Scalebar is 100μm.

To put our fluorescent trimeric RBD proteins to the test we stained ferret lung tissue slides together with the ACE2 antibody to determine co-localization (Fig 6A). Indeed ACE2 binding by the antibody was almost completely overlapping compared with the trimeric, fully glycosylated, RBD proteins. As we do not observe any competition it is clear that the antibody and RBD proteins target different epitopes.

We analyzed SARS-CoV-2 RBD binding to Syrian hamster lung tissues before and after 4-days post-infection (dpi) to determine possible infection-mediated downregulation of ACE2. The trimeric RBD protein bound to mock-infected Syrian hamster lung tissue slides but failed to bind at 4dpi. We also stained these sections with an anti-nucleoprotein (NP) antibody to determine where infection took place and the anti ACE2 antibody. The NP antibody failed to bind to mock-infected lung tissue as expected, while it did bind to the infected lung tissue, demonstrating distinct foci. Finally, the ACE2 antibody bound to Syrian hamster lung tissue 4dpi, yet at a diminished rate compared to mock infected. Thus both SARS-CoV RBD trimers fail to bind to infected lung tissues, whereas, although downregulated, ACE2 is present.

## Discussion

In this report, we describe the generation of fluorescent trimeric RBD proteins derived from SARS-CoV-1 and 2. These proteins can be efficiently expressed in adherent cells and purified directly from the cell culture supernatant using the increased protein yields provided by the introduction of a C-terminal sfGFP or mOrange fusion. The fluorescent RBD trimers were superior in receptor binding on tissues compared to their monomeric RBD counterparts.

Mature N-glycosylation appears to be important for the avid binding of trimeric RBD proteins to ACE2 in multiple different assays. There is a wealth of information on site-specific N- and O- glycan conformations on spike proteins [32–34], with some disagreement on the nature of the RBD N-glycans. In our protein preparations, they appear to be complex as the majority could not be cleaved by EndoH (S1 Fig). Furthermore, the N-glycosylation of the NTD domain has been shown to affect the conformation of the RBD in full-length spikes [35], however similar analyses on the RBD N-glycans are lacking.

Currently, most knowledge of ACE2 expression is based on genomic and transcriptomic data [36]. However, these analyses are limited as it does not determine expression biochemically on epithelial cells that make contact to the outside world [37]. Several groups have transfected variants of ACE2 in cells to analyze transduction by SARS-CoV-pseudo viruses, although informative these studies do not provide information on the natural expression pattern of ACE2 in susceptible hosts [38,39]. A detailed understanding of the difference between animal species and cell-specific expression of ACE2 at the molecular level is essential, as this can provide valuable knowledge on potential hosts that can be susceptible to SARS-like coronaviruses. An intriguing observation we made is that cells, that do not support efficient replication, are bound by SARS-CoV RBD proteins, in contrast to mouse lung tissues in which ACE2 was not a receptor. Important as well, are the apparent differences between commercially available antibodies. Finally, we demonstrate that our fluorescent trimeric RBD proteins are complementary to study ACE2 expression and SARS-CoV receptor binding dynamics.

An intriguing observation was the abundant expression of ACE2 in terminal bronchioles both in the ferret and Syrian hamster lungs, which are used as experimental animal models. SARS-CoV-RBD protein binding correlated perfectly with the staining of the ACE2 antibody and a ferret infection study [25]. In another ferret infection study, however, the infection was observed in nasal epithelium and many type I pneumocytes and fewer type II pneumocytes in the lungs, with few bronchial epithelial cells expressing viral antigen [26]. Some studies have been performed with anti-ACE2 antibodies, but these stainings appear not to correlate with infection patterns [40,41]. We observed a similar trend indicating that several other factors may be important to infect cells and that infection could, in turn, regulate ACE2 expression.

## Material and methods

### Ethics statement

Animals were handled in an ABSL3 biocontainment laboratory. Research was conducted in compliance with the Dutch legislation for the protection of animals used for scientific purposes (2014, implementing EU Directive 2010/63) and other relevant regulations. The licensed establishment where this research was conducted (Erasmus MC) has an approved OLAW Assurance # A5051-01. Research was conducted under a project license from the Dutch competent authority and the study protocol (#17–4312) was approved by the institutional Animal Welfare Body. Animals were housed in groups of 2 animals in Class III isolators allowing social interactions, under controlled conditions of humidity, temperature and light (12-hour light/12-hour dark cycles). Food and water were available ad libitum. Animals were cared for and monitored (pre- and post-infection) by qualified personnel. The animals were sedated/anesthetized for all invasive procedures.

### Expression and purification of coronavirus spike proteins for binding studies

Recombinant SARS-CoV-1 and -2 envelope proteins and their subunits were cloned using Gibson assembly from cDNAs encoding codon-optimized open reading frames of full-length SARS-CoV-1 and -2 spikes (A kind gift of Rogier Sanders, Amsterdam Medical Centre, The Netherlands). The pCD5 expression vector as described previously was adapted to clone the SARS-1 (GenBank: MN908947.3) and 2 (GenBank: MN908947.3), ectodomains (SARS-2 15–1213, SARS-1 15–1195), N-terminal S1 (SARS-2 15–318, SARS-1 15–305, M41 19 to 272) and RBDs (SARS-2 319–541, SARS-1 306–527) sequences coding for a secretion signal sequence, a GCN4 trimerization domain (RMKQIEDKIEEIESKQKKIENEIARIKK) followed by a seven amino acid cleavage recognition sequence (ENLYFQG) of tobacco etch virus (TEV), a super folder GFP [13], or mOrange2 [42] and the Twin-Strep (WSHPQFEKGGGSGGGSWSHPQFEK); IBA, Germany). Alongside we expressed infectious bronchitis virus M41 spike RBD (GenBank: AY851295.1) [43],) and influenza A virus PR8 HA (GenBank: NP_040980) [13]. The viral envelope proteins were purified from cell culture supernatants after expression in HEK293T or HEK293GnTI^-/-^ cells as described previously [44]. In short, transfection was performed using the pCD5 expression vectors and polyethyleneimine I. The transfection mixtures were replaced at 6 h post-transfection by 293 SFM II expression medium (Gibco), supplemented with sodium bicarbonate (3.7 g/L), Primatone RL-UF (3.0 g/L), glucose (2.0 g/L), glutaMAX (Gibco), valproic acid (0,4 g/L) and DMSO (1,5%). At 5 to 6 days after transfection, tissue culture supernatants were collected.

For ACE2 inhibition studies, ACE2 was expressed in a highly identical fashion, ACE2 (Addgene Plasmid #145171) was cloned into a pCD5 expression vector with SacI and BamHI restriction enzymes. The adapted pCD5 expression vector with an N-terminal HA leader (MKTIIALSYIFCLVFA) peptide, ACE2, and Twin-Strep (WSHPQFEKGGGSGGGSWSHPQFEK); IBA, Germany) was purified from HEK293T cell culture supernatant.

### Determining expression yield

We measure fluorescence in the cell culture supernatant when applicable using a polarstar fluorescent reader with excitation and emission wavelengths of 480 nm and 520 nm for sfGFP and 520nm and 550nm for mOrange2, respectively. Spike protein expression was confirmed by western blotting using a StrepMAB-HRP classic antibody. Proteins are purified using a single-step with strepTactin sepharose beads in batch format. Purified proteins were pre-treated (were indicated) with PNGase F or EndoH (New England Biolabs, USA) according to the manufacturer’s protocol before analysis by Western blotting using Strep-MAb-HRP classic antibody.

### Antigen ELISA

Plates were coated with 2 μg/mL spike protein in PBS for 16 hours at 4°C, followed by blocking by 3% bovine serum albumin (BSA, VWR, 421501J) in phosphate-buffered saline-Tween 0,1% (PBS-T 0,1%). After the block, the proteins were detected using a 21-days post-infection macaque serum and serum of the same monkey before infection. Serum starting dilution was 1:100 and diluted 1:1 for 5 times and incubated at RT for 2 hrs. Next, wells were treated with goat-α-human HRP secondary antibody for 1 hour at room temperature. Serum spike binding antibodies were detected using ODP and measured in a plate reader (Polarstar Omega, BMG Labtech) at 490 nm.

### Negative stain electron microscopy structural analysis

SARS-CoV2-RBD-GCN4-sfGFP in 10mM Tris, 150mM NaCl at 4°C was deposited on 400 mesh copper negative stain grids and stained with 2% uranyl formate. The grid was imaged on a 200KeV Tecnai F20 electron microscope and a 4k x 4k TemCam F416 camera. Micrographs were collected using Leginon [45] and then uploaded to Appion [46]. Particles were picked using DoGPicker [47], stacked. 2D processing was undertaken using Relion. Images showing Trimeric RBD, a GCN4 connector, and three sfGFPs.

### Immunofluorescent cell staining

VERO, A549, and MDCK cells grown on coverslips were analyzed by immunofluorescent staining. Cells were fixed with 4% paraformaldehyde in PBS for 25 min at RT after which permeabilization was performed using 0,1% Triton in PBS. Subsequently, the coronavirus spike proteins were applied at 50μg/ml for 1 h at RT. Primary Strep-MAb classic chromeo-488 (IBA) and secondary Alexa-fluor 488 or 555 goat anti-mouse (Invitrogen) were applied sequentially with PBS washes in between. DAPI (Invitrogen) was used as nuclear staining. Samples were imaged on a Leica DMi8 confocal microscope equipped with a 10x HC PL Apo CS2 objective (NA. 0.40). Excitation was achieved with a Diode 405 or white light for excitation of Alexa488 and Alexa555, a pulsed white laser (80MHz) was used at 488 nm and 549 nm, and emissions were obtained in the range of 498-531nm and 594–627 nm respectively. Laser powers were 10–20% with a gain of a maximum of 200. LAS Application Suite X was used as well as ImageJ for the addition of the scale bars.

### Tissue staining

Serial sections of formalin-fixed, paraffin-embedded ferret, Syrian hamster, and mouse lungs were obtained from the Department of Veterinary Pathobiology, Faculty of Veterinary Medicine, Utrecht University, and the department of Viroscience, Erasmus University, The Netherlands, respectively. All relevant ethical regulations for the use of animal tissues have been complied with. Tissue deparaffinized in xylene, rehydrated in a series of alcohol from 100%, 96% to 70%, and lastly in distilled water. Tissue slides were boiled in citrate buffer pH 6.0 for 10 min at 900 kW in a microwave for antigen retrieval and washed in PBS-T three times. Endogenous peroxidase activity was blocked with 1% hydrogen peroxide for 30 min. Tissues were subsequently incubated with 3% BSA in PBS-T overnight at 4°C. The next day, purified viral spike proteins (50μg/ml) or antibodies were added to the tissues for 1 h at RT. With rigorous washing steps in between the secondary antibodies were applied for 45min at RT. Where indicated recombinant RBD and ACE2 proteins were pre-incubated O/N at 4°C before application on lung tissues or cells. For histological staining, HRPO labeled secondary antibodies were used, and binding was visualized using 3-amino-9-ethyl carbazole (AEC) (Sigma-Aldrich) and counterstain was performed using Hematoxylin.

antibodies:

anti ACE2 (abcam 272690) 5ug/mL

anti ACE2 (abcam 15348) 5ug/mL

anti ACE2 (R&D systems AF933) 5ug/mL

anti SARS-CoV-2 nucleoprotein (abcam 40143-R019) 5ug/mL

Primary Strep-MAb classic chromeo-488 or 555 (IBA) 1ug/mL

Secondary goat anti mouse 488 or 555 (Invitrogen) 2ug/mL

Secondary goat anti mouse HRPO (Invitrogen) 2ug/mL

Secondary goat anti rabbit 488 (Thermo A-11008) 2ug/mL

Secondary donkey anti goat 555 (Thermo A-21432) 2ug/mL

### Quantifying and plotting fluorescent image data

Image quantification was performed by measuring the intensity of gray pixels of the stained ferret and Syrian hamster lung tissue slides with ImageJ version 1.52p. For stained tissues, background correction was performed by subtracting the average signal intensity of the antibody control from the images stained with ACE2 antibody, SARS-CoV1, and SARS-CoV2 recombinant proteins. Regions of interest were set by highlighting the area using the Image -> Adjust -> Threshold setting. The 8-bit images were checked for saturation by plotting the distribution of gray values with Analyze -> Histogram. Subsequent analysis was performed by quantifying the mean ± standard deviation of alveoli/cells stained with distinct proteins in various images (Analyze -> Measure). Plotting means ± standard deviation values were performed in GraphPad Prism v8.0.1. A 3D Surface plot was generated for several staining conditions with Analyze -> 3D Surface Plot.

### Sample preparation for glycoproteomics analysis

7 μg of EndoH (NEB, New England BioLabs) treated and untreated SARS-CoV-1 and SARS-CoV-2 RBD domains (monomeric and trimeric) were digested following the S-trap Micro spin-column protocol (Protifi). Briefly, samples were mixed in the ratio 1:1 (v/v) with 10% SDS, 100 mM triethylammonium bicarbonate (TEAB), pH 7.55. Next, samples were reduced with 20 mM DTT (final concentration) at 55°C for 15 minutes, followed by alkylation with 40 mM iodoacetamide (final concentration) for 30 minutes in the dark. Then, samples were acidified with 12% phosphoric acid at the ratio 10:1 (v/v) respectively and mixed with the S-trap binding buffer (90% methanol, 100 mM TEAB, pH 7.1) in the ratio 1:6 (v/v) respectively. The resulting sample mixture was added to the S-trap spin column and centrifuged at 4000 × *g* for 2 min at room temperature. The captured proteins were washed six times with S-trap binding buffer. Following washing, 25 μL of GluC protease (Sigma Aldrich) in 25 mM ammonium bicarbonate (1:30 w/w ratio) were added to the S-trap column and incubated at 37°C for 3 hours. Next, 25 μL of trypsin (Promega) in 25 mM ammonium bicarbonate (1:30 w/w ratio) were applied to the same S-trap column and incubated for 2 hours at 37°C. After that, 150 μL of ammonium bicarbonate was added to the S-trap and incubated overnight at 37°C. Finally, the peptides were eluted from the S-trap in 3 steps by centrifugation at 4000 × *g* for 2 min: first elution of ammonium bicarbonate-peptide mixture, followed by elution with 40 μL of 0.2% formic acid and, lastly, with 40 μL of 50% acetonitrile/0.1% formic acid (v/v). All elutions we pooled, vacuum-dried, and resuspended in 85 μL of 2% formic acid.

### Mass spectrometry

5 μl of resuspended peptides were analyzed on an Orbitrap Fusion Tribrid (ThermoFisher Scientific, Bremen) mass spectrometer coupled to nanospray UHPLC system Ultimate3000 (ThermoFisher) in duplicates. A 90-min LC gradient from 0% to 35% acetonitrile was used to separate peptides at a flow rate of 300 ml/min. A Poroshell 120 EC C18 (50 cm × 75 μm, 2.7 μm, Agilent Technologies) analytical column and an Acclaim Pepmap 100 C18 (5 mm × 0.3 mm, 5 μm, ThermoFisher Scientific) trap column was used for the peptide separation. The data were acquired in data-dependent mode. Orbitrap Fusion parameters for the full scan MS spectra were as follows: an AGC target of 4 × 10^5^ at 60,000 resolution, scan range 350–2000 *m/z*, Orbitrap maximum injection time 50 ms. Ten most intense ions (2+ to 8+ ions) were subjected to fragmentation with higher energy collision dissociation set to 30%. Once oxonium ions corresponding to the glycan fragmentation were detected in MS2 spectra, the same precursor ions were subjected to an electron-transfer/higher-energy collision dissociation ion fragmentation scheme. The supplemental higher-energy collision dissociation energy was set at 27%. The MS2 spectra were acquired at a resolution of 30,000 with an AGC target of 5∗10^5^, maximum injection time 250 ms, scan range 120–4000 *m/z*, and dynamic exclusion of 16 s.

### Mass spectrometry data analysis

The acquired data were searched for glycan modifications with Byonic against a custom database of recombinant RBD domains of SARS-CoV-1 and SARS-CoV-2 and proteases. The search window was set to 12/24 ppm for MS1/MS2, respectively, and a False Discovery Rate (FDR) to 1%. Up to five missed cleavages were allowed using C-terminal cleavage at R/K/E/D to account for the sequential GluC-trypsin digestion. Carbamidomethylation of cysteine was set as a fixed modification, methionine oxidation as variable common 1, glycan modifications as variable common 2, permitting up to max. 2 variable common modifications per one peptide. A glycan database containing 305 *N*-linked glycans was used in the search. Glycopeptide hits reported in the Byonic results file were initially accepted if the Byonic score was ≥200, LogProb ≥2, and peptide length was at least 6 amino acids. Accepted glycopeptides were manually inspected for the quality of fragment assignments. The glycopeptide was considered true-positive if the appropriate b, y, c, and z fragment ions were matched in the spectrum, as well as the corresponding oxonium ions of the identified glycans. All glycopeptide identifications were merged into a single non-redundant list per sequon. Glycans were classified based on HexNAc content as chitobiose (up to maximum 2 HexNAc and 1 Fuc), high-mannose (2 HexNAc), hybrid (3 HexNAc), or complex (>3 HexNAc). Byonic search results were exported to mzIdentML format. These files were used to build a spectral library in Skyline and extract peak areas for individual glycoforms from MS1 scans. The full database of variable *N*-linked glycan modifications from Byonic was manually added to the Skyline project file in XML format. Glycopeptide identifications from Byonic were manually inspected in Skyline and evaluated for correct isotope assignments and well-defined elution profiles, acceptable for peak integration. In the case of multiple missed cleavages, reporting on the same site-specific glycoform, peak areas were summed in the semi-quantitative analysis. Reported peak areas were pooled based on the number of HexNAc residues to distinguish chitobiose/high-mannose/hybrid/complex glycosylation. The semi-quantitative analysis of the glycosylation profile was performed per site. The acquired quantitative data were illustrated with GraphPad Prism 8 software.

## Supporting information

S1 Fig Glycosylation analyses of the SARS-CoV protein preparations. **(A) PNGAse F and EndoH treatment of SARS-CoV-1 RBD proteins.** 0.5μg of protein was subjected without or with PNGase F or EndoH for 1hr and subjected to SDS-PAGE and western blot analyzes. **(B) PNGAse F and EndoH treatment of SARS-CoV-2 RBD proteins.** As in (A)**. (A) PNGAse F and EndoH treatment of SARS-CoV-2 hexapro full-length ectodomain trimer from 293T cells and the M41NTD trimer from both 293T and GnTI**^**-/-**^ **cells.** 0.5μg of protein was subjected without or with PNGase F or EndoH for 1hr and subjected to SDS-PAGE and western blot analyzes. **(D) Semi-quantitative glycoproteomic analysis of N-linked glycosylation of SARS-CoV-1 and SARS-CoV-2 trimeric and monomeric RBDs.** Semi-quantitative analyses are based on extracted peak areas of site-specific N-glycosylation and represented by glycan type (chitobiose, high-mannose, and complex/hybrid). Chitobiose type refers to the glycans consisting of only one or two HexNAc residues and/or one fucose. High-mannose type refers to the glycans with a maximum of 2 HexNAc residues extended with oligomannoses (2–8 mannoses) and/or fucose. Complex/Hybrid type refers to the glycans with at least 3 HexNAc residues and extended with various monosaccharide residues. Error bars represent the standard deviation of the duplicate measurement. A full overview is presented in S1 Data.
(TIF)

S2 Fig Binding of SARS-COV spike protein domains to cell lines to A549, VERO, and MDCK cells. Proteins were applied 50μg/ml and were detected using anti-strep and goat-anti-mouse antibodies. Scalebar is 5μm.
(TIF)

S3 Fig Comparison of 2 anti-ACE2 antibodies from Abcam. Cells and tissue were stained with 5ug/ml of the designated antibody for 1 hr and detected after several washing steps for 1hr with an goat-anti-rabbit-alexa488 or a donkey-anti-goat-alexa555. Scalebar is 100μm.
(TIF)

S4 Fig Binding of RBD proteins to tissues. **(A) Binding of RBD proteins fused to mOrange2 to ferret lung tissues.** Proteins were applied 50μg/ml and detected using anti-strep and goat-anti-mouse antibodies. Scalebar is 100μm. **(B) Binding of RBD proteins fused to sfGFP proteins to ferret lung tissues, using HRP as a readout.** Identical experiment to (A) but using an HRP readout using anti-strep and goat-anti-mouse antibodies. Scalebar is 100μm. **(C) Control staining on ferret lung tissues using HRP as readout.** M41, NTD trimers of SARS-CoV-1 and -2 and antibodies only. Proteins were applied 50μg/ml and detected using anti-strep and goat-anti-mouse antibodies. Scalebar is 100μm. **(D) Lack of NTD binding to ferret lung tissue using fluorescence.** Proteins were applied 50μg/ml and detected using anti-strep and goat-anti-mouse antibodies. Scalebar is 100μm. **(E) Control stainings to Syrian hamster tissues, antibodies only and M41.** Proteins were applied 50μg/ml and where indicated pre-incubated with recombinant ACE2 protein. Scalebar is 100μm.
(TIF)

S1 Data Raw data of the N-glycan Mass spectrometry measurements. (XLSX)

## References

[1] Chen X, Li R, Pan Z, Qian C, Yang Y, et al Human monoclonal antibodies block the binding of SARS-CoV-2 spike protein to angiotensin converting enzyme 2 receptor. Cell Mol Immunol. 2020 10.1038/s41423-020-0426-7 32313207 PMC7167496

[2] Wang Q, Zhang Y, Wu L, Niu S, Song C, et al Structural and Functional Basis of SARS-CoV-2 Entry by Using Human ACE2. Cell. 2020 10.1016/j.cell.2020.03.045 32275855 PMC7144619

[3] Letko M, Marzi A, Munster V. Functional assessment of cell entry and receptor usage for SARS-CoV-2 and other lineage B betacoronaviruses. Nature Micro. 2020;5:562–569. 10.1038/s41564-020-0688-y 32094589 PMC7095430

[4] Walls AC, Park YJ, Tortorici MA, Wall A, McGuire AT, et al Structure, Function, and Antigenicity of the SARS-CoV-2 Spike Glycoprotein. Cell. 2020;181:281–292 e286. 10.1016/j.cell.2020.02.058 32155444 PMC7102599

[5] Amanat F, Stadlbauer D, Strohmeier S, Nguyen THO, Chromikova V, et al A serological assay to detect SARS-CoV-2 seroconversion in humans. Nature Med. 2020 10.1101/2020.03.17.20037713 32398876 PMC8183627

[6] Yuan M, Wu NC, Zhu X, Lee CD, So RTY, et al A highly conserved cryptic epitope in the receptor-binding domains of SARS-CoV-2 and SARS-CoV. Science. 2020 10.1126/science.abb7269 32245784 PMC7164391

[7] Brouwer PJM, Caniels TG, van der Straten K, Snitselaar JL, Aldon Y, et al Potent neutralizing antibodies from COVID-19 patients define multiple targets of vulnerability. Science. 2020 10.1126/science.abc5902 32540902 PMC7299281

[8] Xu C, Wang Y, Liu C, Zhang C, Han W, et al Conformational dynamics of SARS-CoV-2 trimeric spike glycoprotein in complex with receptor ACE2 revealed by cryo-EM. bioRxiv. 10.1126/sciadv.abe5575 2020;2020.2006.2030.177097. 33277323 PMC7775788

[9] McCallum M, Walls AC, Corti D, Veesler D. Closing coronavirus spike glycoproteins by structure-guided design. bioRxiv. 2020: 2020.2006.2003.129817. 10.1101/2020.06.03.129817 32577661 PMC7302216

[10] Ke Z, Oton J, Qu K, Cortese M, Zila V, et al Structures, conformations and distributions of SARS-CoV-2 spike protein trimers on intact virions. bioRxiv. 2020: 2020.2006.2027.174979. 10.1038/s41586-020-2665-2 32805734 PMC7116492

[11] Juraszek J, Rutten L, Blokland S, Bouchier P, Voorzaat R, et al Stabilizing the Closed SARS-CoV-2 Spike Trimer. bioRxiv. 2020: 2020.2007.2010.197814. 10.1038/s41467-020-20321-x 33431842 PMC7801441

[12] Hsieh C-L, Goldsmith JA, Schaub JM, DiVenere AM, Kuo H-C, et al Structure-based Design of Prefusion-stabilized SARS-CoV-2 Spikes. bioRxiv. 2020: 2020.2005.2030.125484. 10.1101/2020.05.30.125484 32703906 PMC7402631

[13] Nemanichvili N, Tomris I, Turner HL, McBride R, Grant OC, et al Fluorescent Trimeric Hemagglutinins Reveal Multivalent Receptor Binding Properties. J Mol Biol. 2019;431:842–856. 10.1016/j.jmb.2018.12.014 30597163 PMC6397626

[14] de Vries RP, de Vries E, Bosch BJ, de Groot RJ, Rottier PJ, et al The influenza A virus hemagglutinin glycosylation state affects receptor-binding specificity. Virology. 2010;403:17–25. 10.1016/j.virol.2010.03.047 20441997

[15] de Vries RP, Smit CH, de Bruin E, Rigter A, de Vries E, et al (2012) Glycan-dependent immunogenicity of recombinant soluble trimeric hemagglutinin. J Virol 86: 11735–11744. 10.1128/JVI.01084-12 22915811 PMC3486279

[16] Reeves PJ, Callewaert N, Contreras R, Khorana HG (2002) Structure and function in rhodopsin: High-level expression of rhodopsin with restricted and homogeneous N-glycosylation by a tetracycline-inducible N-acetylglucosaminyltransferase I-negative HEK293S stable mammalian cell line. PNAS 99: 13419–13424. 10.1073/pnas.212519299 12370423 PMC129688

[17] Hsieh CL, Goldsmith JA, Schaub JM, DiVenere AM, Kuo HC, et al Structure-based design of prefusion-stabilized SARS-CoV-2 spikes. Science. 2020. 10.1126/science.abd0826 PMC7402631 32703906

[18] Frese CK, Altelaar AF, van den Toorn H, Nolting D, Griep-Raming J, et al Toward full peptide sequence coverage by dual fragmentation combining electron-transfer and higher-energy collision dissociation tandem mass spectrometry. Anal Chem. 2012;84:9668–9673. 10.1021/ac3025366 23106539

[19] Rockx B, Kuiken T, Herfst S, Bestebroer T, Lamers MM, et al Comparative pathogenesis of COVID-19, MERS, and SARS in a nonhuman primate model. Science. 2020 10.1126/science.abb7314 32303590 PMC7164679

[20] Yuan M, Liu H, Wu NC, Lee C-CD, Zhu X, et al Structural basis of a shared antibody response to SARS-CoV-2. Science. 2020: eabd2321 10.1126/science.abd2321 32661058 PMC7402627

[21] Harcourt J, Tamin A, Lu X, Kamili S, Sakthivel SK, et al Severe Acute Respiratory Syndrome Coronavirus 2 from Patient with 2019 Novel Coronavirus Disease, United States. Emerg Infect Dis. 2020;26 10.3201/eid2606.200516 32160149 PMC7258473

[22] Ge XY, Li JL, Yang XL, Chmura AA, Zhu G, et al Isolation and characterization of a bat SARS-like coronavirus that uses the ACE2 receptor. Nature. 2013;503:535–538. 10.1038/nature12711 24172901 PMC5389864

[23] Yang XL, Hu B, Wang B, Wang MN, Zhang Q, et al Isolation and Characterization of a Novel Bat Coronavirus Closely Related to the Direct Progenitor of Severe Acute Respiratory Syndrome Coronavirus. J Virol. 2015;90:3253–3256. 10.1128/JVI.02582-15 26719272 PMC4810638

[24] Li Q, Wu J, Nie J, Zhang L, Hao H, et al The Impact of Mutations in SARS-CoV-2 Spike on Viral Infectivity and Antigenicity. Cell. 2020;182:1284–1294.e1289. 10.1016/j.cell.2020.07.012 32730807 PMC7366990

[25] Kim Y-I, Kim S-G, Kim S-M, Kim E-H, Park S-J, et al Infection and Rapid Transmission of SARS-CoV-2 in Ferrets. Cell Host & Microbe. 2020;27:704–709.e702. 10.1016/j.chom.2020.03.023 32259477 PMC7144857

[26] Richard M, Kok A, de Meulder D, Bestebroer TM, Lamers MM, et al SARS-CoV-2 is transmitted via contact and via the air between ferrets. Nat Commun. 2020;11:3496 10.1038/s41467-020-17367-2 32641684 PMC7343828

[27] Oreshkova N, Molenaar RJ, Vreman S, Harders F, Oude Munnink BB, et al SARS-CoV-2 infection in farmed minks, the Netherlands, April and May 2020. Euro Surveill. 2020;25 10.2807/1560-7917.ES.2020.25.23.2001005 32553059 PMC7403642

[28] McCray PB, Pewe L, Wohlford-Lenane C, Hickey M, Manzel L, et al Lethal infection of K18-hACE2 mice infected with severe acute respiratory syndrome coronavirus. Journal of Virology. 2007;81:813–821. 10.1128/JVI.02012-06 17079315 PMC1797474

[29] Imai M, Iwatsuki-Horimoto K, Hatta M, Loeber S, Halfmann PJ, et al Syrian hamsters as a small animal model for SARS-CoV-2 infection and countermeasure development. Proceedings of the National Academy of Sciences. 2020;117:16587–16595. 10.1073/pnas.2009799117 32571934 PMC7368255

[30] Roberts A, Vogel L, Guarner J, Hayes N, Murphy B, et al Severe Acute Respiratory Syndrome Coronavirus Infection of Golden Syrian Hamsters. J Virol. 2005;79:503–511. 10.1128/JVI.79.1.503-511.2005 15596843 PMC538722

[31] Sia SF, Yan L-M, Chin AWH, Fung K, Choy K-T, et al Pathogenesis and transmission of SARS-CoV-2 in golden hamsters. Nature. 2020;583:834–838. 10.1038/s41586-020-2342-5 32408338 PMC7394720

[32] Shajahan A, Supekar NT, Gleinich AS, Azadi P. Deducing the N- and O- glycosylation profile of the spike protein of novel coronavirus SARS-CoV-2. Glycobiology. 2020 10.1093/glycob/cwaa042 32363391 PMC7239183

[33] Watanabe Y, Allen JD, Wrapp D, McLellan JS, Crispin M. Site-specific glycan analysis of the SARS-CoV-2 spike. Science. 2020;369:330–333. 10.1126/science.abb9983 32366695 PMC7199903

[34] Zhang Y, Zhao W, Mao Y, Chen Y, Wang S, et al Site-specific N-glycosylation Characterization of Recombinant SARS-CoV-2 Spike Proteins. bioRxiv. 2020: 2020.2003.2028.013276. 10.1074/mcp.RA120.002295 33077685 PMC7876485

[35] Henderson R, Edwards RJ, Mansouri K, Janowska K, Stalls V, et al Glycans on the SARS-CoV-2 Spike Control the Receptor Binding Domain Conformation. bioRxiv. 2020 10.1101/2020.06.26.173765 32637959 PMC7337389

[36] Lukassen S, Chua RL, Trefzer T, Kahn NC, Schneider MA, et al SARS-CoV-2 receptor ACE2 and TMPRSS2 are primarily expressed in bronchial transient secretory cells. EMBO J. 2020: e105114 10.15252/embj.20105114 32246845 PMC7232010

[37] Hikmet F, Méar L, Uhlén M, Lindskog C. The protein expression profile of ACE2 in human tissues. bioRxiv. 2020: 2020.2003.2031.016048. 10.15252/msb.20209610 32715618 PMC7383091

[38] Conceicao C, Thakur N, Human S, Kelly JT, Logan L, et al The SARS-CoV-2 Spike protein has a broad tropism for mammalian ACE2 proteins. bioRxiv. 2020: 2020.2006.2017.156471. 10.1371/journal.pbio.3001016 33347434 PMC7751883

[39] Tang Y-D, Li Y-M, Sun J, Zhang H-L, Wang T-Y, et al Cell entry of SARS-CoV-2 conferred by angiotensin-converting enzyme 2 (ACE2) of different species. bioRxiv. 2020: 2020.2006.2015.153916.

[40] Hikmet F, Mear L, Edvinsson A, Micke P, Uhlen M, et al The protein expression profile of ACE2 in human tissues. bioRxiv. 2020: 2020.2003.2031.016048. 10.15252/msb.20209610 32715618 PMC7383091

[41] Hamming I, Timens W, Bulthuis ML, Lely AT, Navis G, et al Tissue distribution of ACE2 protein, the functional receptor for SARS coronavirus. A first step in understanding SARS pathogenesis. J Pathol. 2004;203:631–637. 10.1002/path.1570 15141377 PMC7167720

[42] Shaner NC, Lin MZ, McKeown MR, Steinbach PA, Hazelwood KL, et al Improving the photostability of bright monomeric orange and red fluorescent proteins. Nat Methods. 2008;5:545–551. 10.1038/nmeth.1209 18454154 PMC2853173

[43] Bouwman KM, Parsons LM, Berends AJ, de Vries RP, Cipollo JF, et al Three Amino Acid Changes in Avian Coronavirus Spike Protein Allow Binding to Kidney Tissue. J Virol. 2020;94 10.1128/JVI.01363-19 31694947 PMC6955270

[44] van der Woude R, Turner HL, Tomris I, Bouwman KM, Ward AB, et al Drivers of recombinant soluble influenza A virus hemagglutinin and neuraminidase expression in mammalian cells. Protein Sci. 2020 10.1002/pro.3918 32710576 PMC7454420

[45] Potter CS, Chu H, Frey B, Green C, Kisseberth N, et al Leginon: a system for fully automated acquisition of 1000 electron micrographs a day. Ultramicroscopy. 1999;77:153–161. 10.1016/s0304-3991(99)00043-1 10406132

[46] Lander GC, Stagg SM, Voss NR, Cheng A, Fellmann D, et al Appion: An integrated, database-driven pipeline to facilitate EM image processing. J Struc Bio. 2009;166:95–102. 10.1016/j.jsb.2009.01.002 19263523 PMC2775544

[47] Voss NR, Yoshioka CK, Radermacher M, Potter CS, Carragher B. DoG Picker and TiltPicker: Software tools to facilitate particle selection in single particle electron microscopy. J Struc Bio. 2009;166: 205–213. 10.1016/j.jsb.2009.01.004 19374019 PMC2768396

