# Supplementary figures and images for: Multimerization- and glycosylation-dependent receptor binding of SARS-CoV-2 spike proteins
Source: PLoS Pathog. 2021 Feb 8;17(2):e1009282. doi: 10.1371/journal.ppat.1009282 (PMC7895411; doi:10.1371/journal.ppat.1009282)

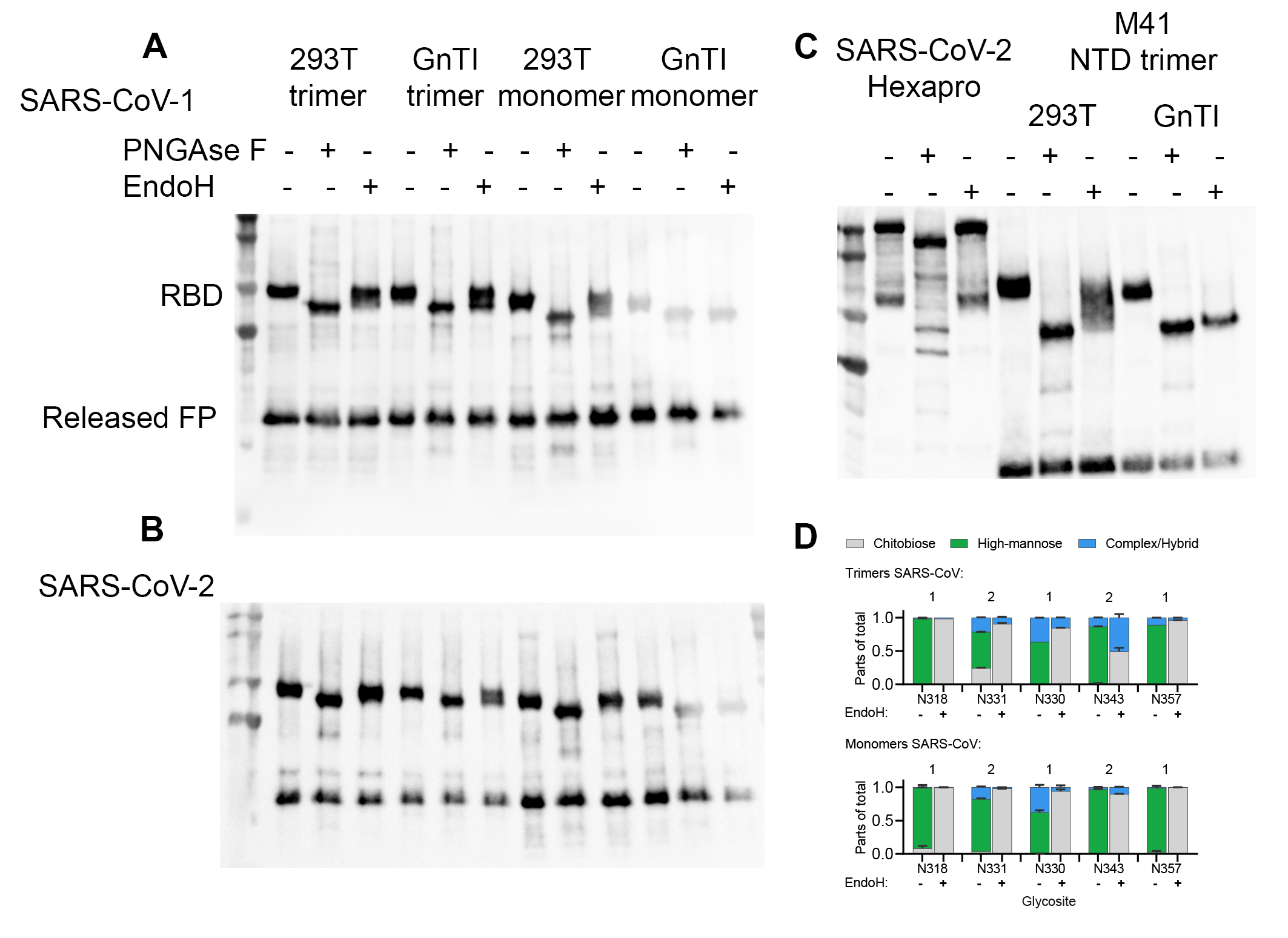

Supplement: S1 Fig — (A) PNGAse F and EndoH treatment of SARS-CoV-1 RBD proteins. 0.5μg of protein was subjected without or with PNGase F or EndoH for 1hr and subjected to SDS-PAGE and western blot analyzes. (B) PNGAse F and EndoH treatment of SARS-CoV-2 RBD proteins. As in (A). (A) PNGAse F and EndoH treatment of SARS-CoV-2 hexapro full-length ectodomain trimer from 293T cells and the M41NTD trimer from both 293T and GnTI-/- cells. 0.5μg of protein was subjected without or with PNGase F or EndoH for 1hr and subjected to SDS-PAGE and western blot analyzes. (D) Semi-quantitative glycoproteomic analysis of N-linked glycosylation of SARS-CoV-1 and SARS-CoV-2 trimeric and monomeric RBDs. Semi-quantitative analyses are based on extracted peak areas of site-specific N-glycosylation and represented by glycan type (chitobiose, high-mannose, and complex/hybrid). Chitobiose type refers to the glycans consisting of only one or two HexNAc residues and/or one fucose. High-mannose type refers to the glycans with a maximum of 2 HexNAc residues extended with oligomannoses (2–8 mannoses) and/or fucose. Complex/Hybrid type refers to the glycans with at least 3 HexNAc residues and extended with various monosaccharide residues. Error bars represent the standard deviation of the duplicate measurement. A full overview is presented in S1 Data. (TIF) [file ppat.1009282.s001.tif]

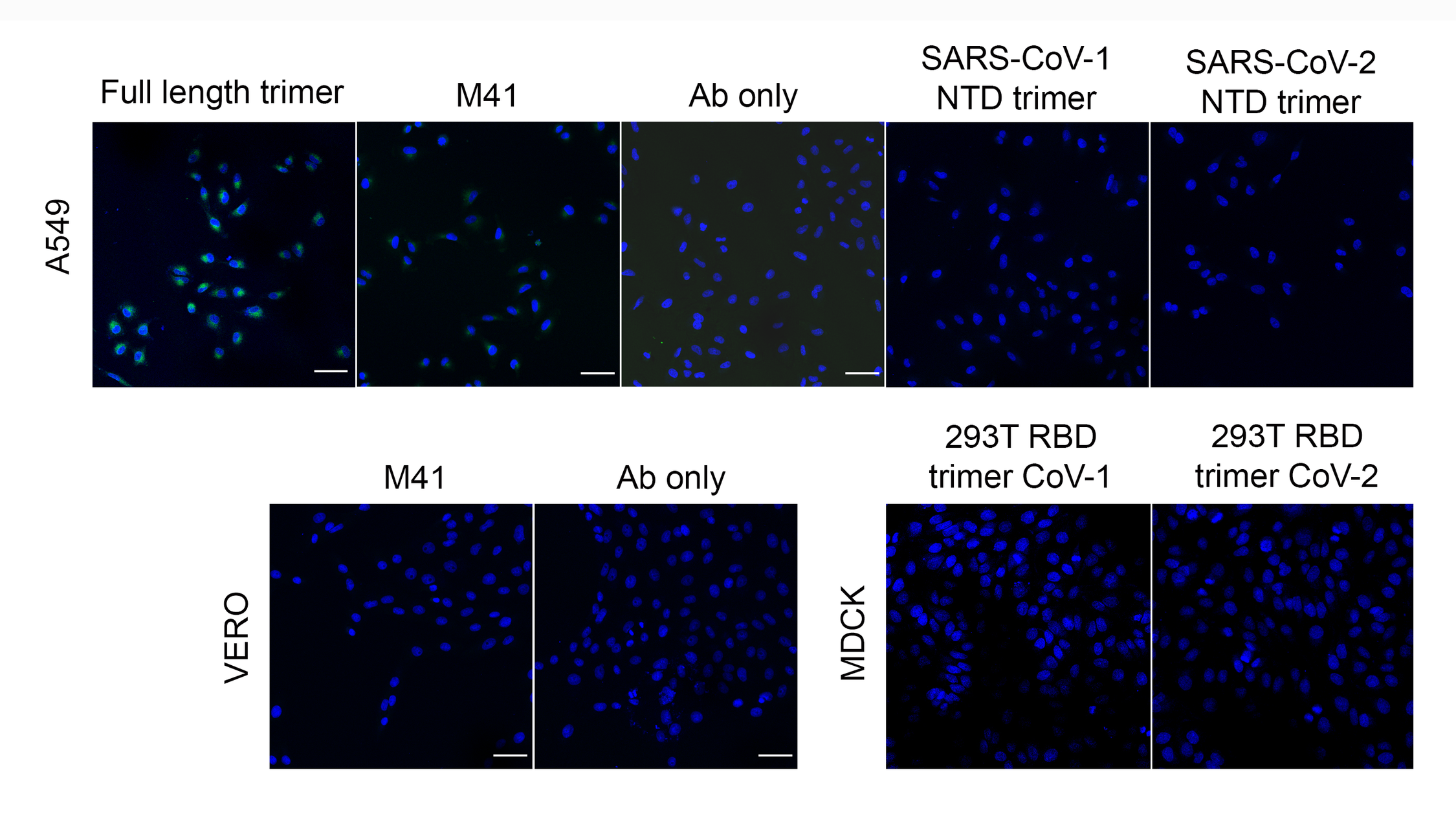

Supplement: S2 Fig — Proteins were applied 50μg/ml and were detected using anti-strep and goat-anti-mouse antibodies. Scalebar is 5μm. (TIF) [file ppat.1009282.s002.tif]

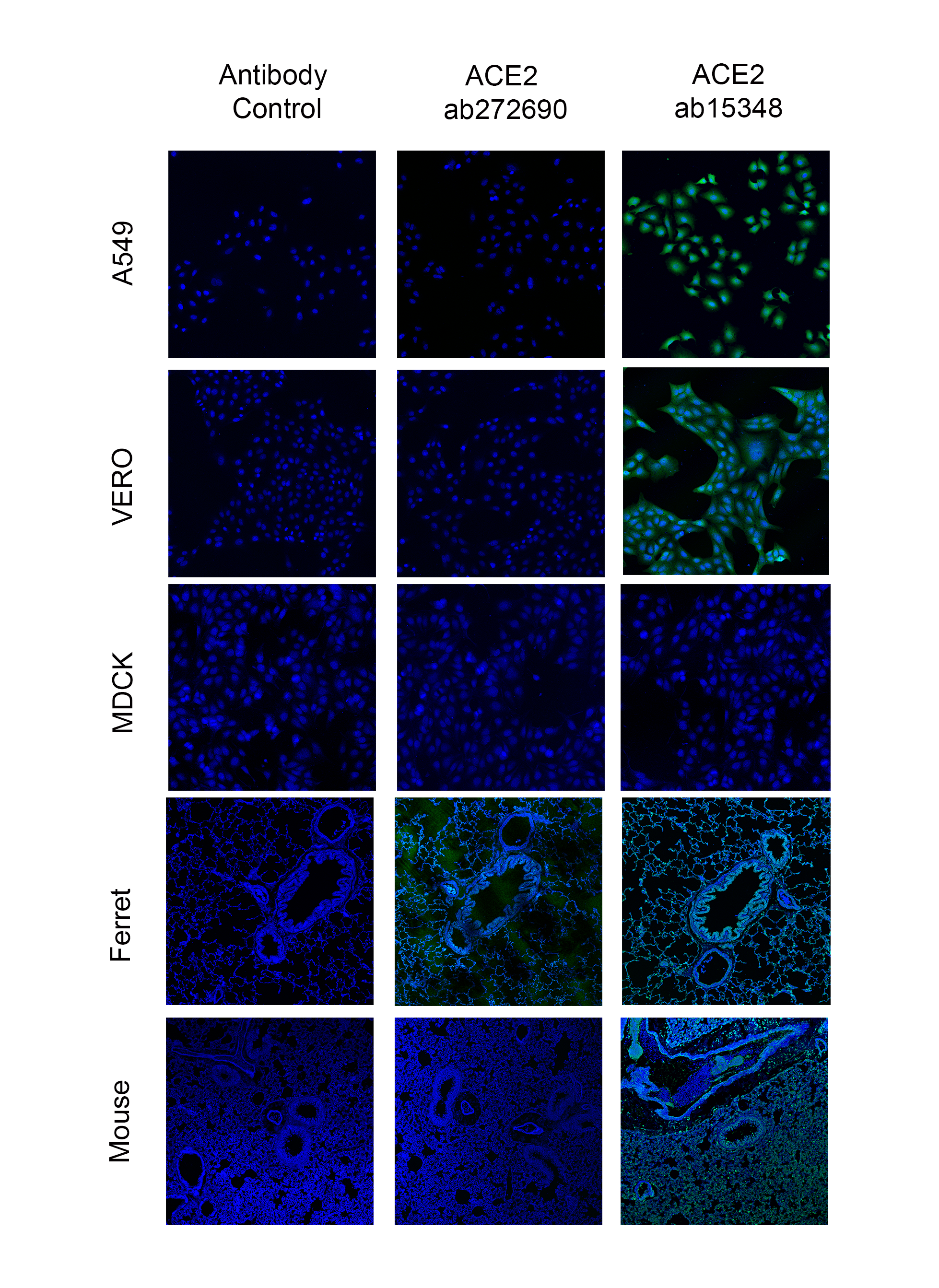

Supplement: S3 Fig — Cells and tissue were stained with 5ug/ml of the designated antibody for 1 hr and detected after several washing steps for 1hr with an goat-anti-rabbit-alexa488 or a donkey-anti-goat-alexa555. Scalebar is 100μm. (TIF) [file ppat.1009282.s003.tif]

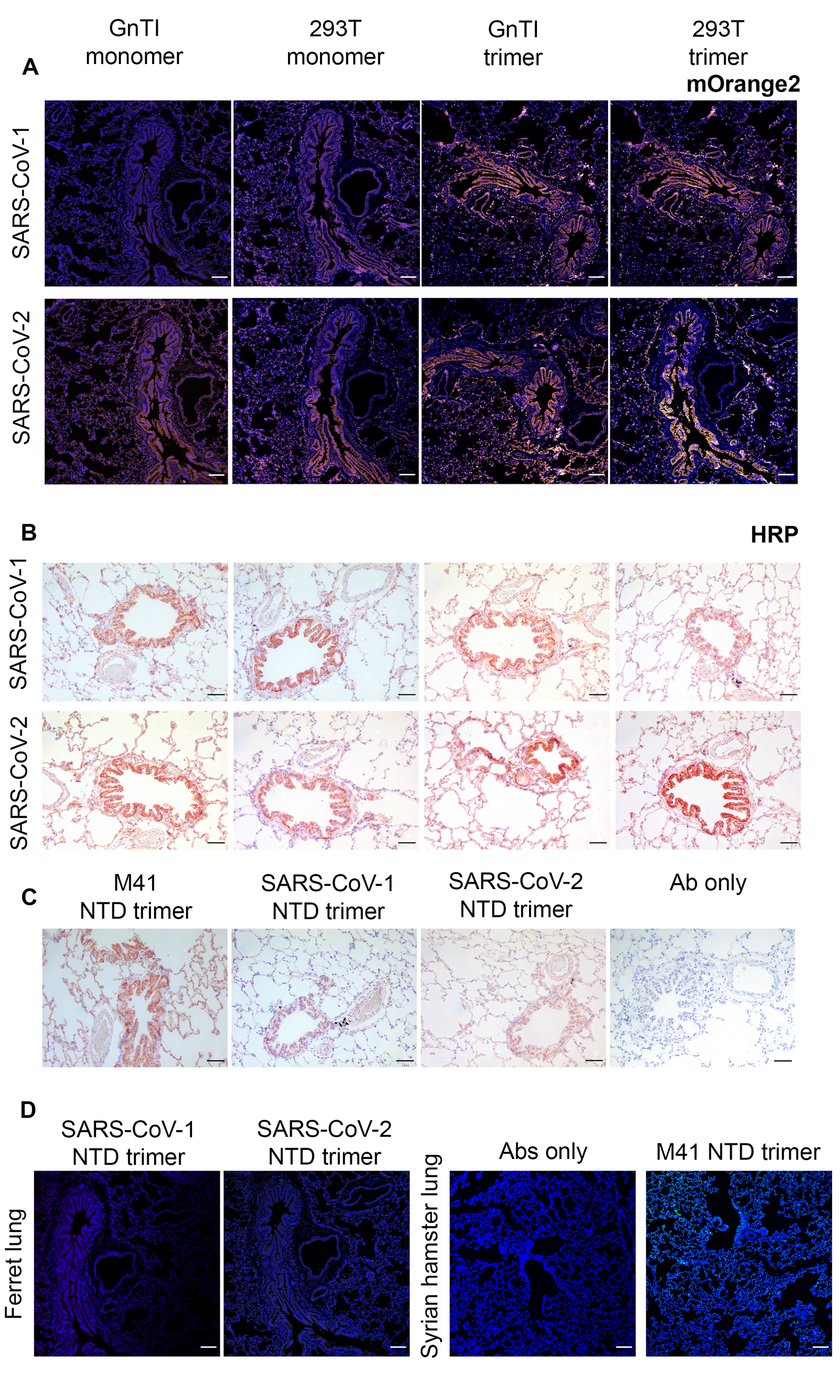

Supplement: S4 Fig — (A) Binding of RBD proteins fused to mOrange2 to ferret lung tissues. Proteins were applied 50μg/ml and detected using anti-strep and goat-anti-mouse antibodies. Scalebar is 100μm. (B) Binding of RBD proteins fused to sfGFP proteins to ferret lung tissues, using HRP as a readout. Identical experiment to (A) but using an HRP readout using anti-strep and goat-anti-mouse antibodies. Scalebar is 100μm. (C) Control staining on ferret lung tissues using HRP as readout. M41, NTD trimers of SARS-CoV-1 and -2 and antibodies only. Proteins were applied 50μg/ml and detected using anti-strep and goat-anti-mouse antibodies. Scalebar is 100μm. (D) Lack of NTD binding to ferret lung tissue using fluorescence. Proteins were applied 50μg/ml and detected using anti-strep and goat-anti-mouse antibodies. Scalebar is 100μm. (E) Control stainings to Syrian hamster tissues, antibodies only and M41. Proteins were applied 50μg/ml and where indicated pre-incubated with recombinant ACE2 protein. Scalebar is 100μm. (TIF) [file ppat.1009282.s004.tif]
